# Atrophy in subcortical gray matter in adult patients with moyamoya disease

**DOI:** 10.1007/s10072-022-06583-x

**Published:** 2023-01-09

**Authors:** Zhiwei Zuo, Guo Li, Ya Chen, Penggang Qiao, Jing Zhu, Peng Wang, Fa Wu, Hongmei Yu, Yalan Jiang, Jindou Yang, Gongjie Li, Rui Jiang, Feizhou Du

**Affiliations:** 1 Department of Radiology, The General Hospital of Western Theater Command, 270# Tianhui Road, Rongdu Avenue, Chengdu, 610000 People’s Republic of China; 2 Department of Radiology, Beijing Friendship Hospital, Capital Medical University, Beijing, People’s Republic of China; 3 Department of Radiology, Affiliated Hospital of Academy of Military Medical Sciences, Beijing, People’s Republic of China

**Keywords:** Moyamoya, Subcortical gray matter, Hippocampus, Amygdala, Atrophy

## Abstract

**Background:**

Acute cerebrovascular accidents, long-term hypoperfusion, and/or remote neuronal degeneration may lead to structural alterations in patients with moyamoya disease (MMD). This study sought to comprehensively investigate the distribution characteristics of subcortical gray matter volume and their correlations with angiographic changes in the intracranial artery in patients with MMD.

**Method:**

One hundred forty-two patients with MMD and 142 age- and sex-matched healthy controls underwent 3-dimensional high-resolution structural magnetic resonance imaging. Volumes of subcortical gray matter and subregions of the hippocampus and amygdala were calculated, and the degree of stenosis/occlusion of intracranial arteries in patients with MMD was evaluated on MR angiography.

**Results:**

Volume reductions in the thalamus, caudate, putamen, hippocampus, amygdala, pallidum, and nucleus accumbens were found in patients with MMD. Hippocampal subfields and amygdala subnuclei in patients with MMD showed distinct vulnerability, and morphological alterations in specific subregions were more obvious than in the whole hippocampus/amygdala. Volume loss in several subcortical areas was related to disease duration and intracranial arterial changes.

**Conclusions:**

Our findings revealed structural alteration patterns of subcortical gray matter in MMD. The specific atrophy in subregions of the hippocampus and the amygdala suggested potential cognitive and affective impairments in MMD, which warrants further investigation. Chronic cerebral hemodynamic alterations in MMD may play a pivotal role in morphological changes in subcortical areas.

**Supplementary Information:**

The online version contains supplementary material available at 10.1007/s10072-022-06583-x.

## Introduction

Patients who were diagnosed with moyamoya disease (MMD) typically experienced one or multiple acute cerebrovascular accidents (CVAs), including transient ischemic attacks, cerebral ischemia, infarction, and hemorrhage. Stenosis or occlusion of the anterior, middle, or posterior cerebral artery is correlated with impairments in the corresponding supply territory, resulting in headache, epilepsy, hemiparesis, hemisensory disturbance, speech abnormity, visual dysfunction, and cognitive impairment [1]. However, before these noticeable symptoms appear, the widespread and continuous vascular steno-occlusive changes of the terminal internal carotid artery and gradual compensatory formation of the arterial collateral vascular network at the base of the brain may have silently occurred in patients with MMD for years and already led to cerebral hemodynamic, functional, and anatomical alterations.

Impairment in functional connectivity in patients with symptomatic MMD has been demonstrated in resting-state functional MRI studies [2–4]. With the enhanced awareness of physical examination and advances in noninvasive magnetic resonance methods, the early detection of asymptomatic patients with MMD who have not experienced CVAs is possible [5]. He et al. [6] found that reduced brain network connectivity and cerebral hypoperfusion already existed in patients with asymptomatic MMD, suggesting that brain functions may not only be influenced by stroke or hemorrhages but also by hemodynamic changes, and abnormal cerebral blood flow and neurovascular coupling are crucial factors underlying the functional impairment in patients with normal cerebrovascular reserve. Chronic hemodynamic disturbance may also lead to regional decreases in cortical thickness [7], a reduction in the density of subcortical areas, and abnormalities in white matter [8] in patients with MMD, which might be other underlying causes of functional abnormalities. Furthermore, the vulnerability of brain microstructure may predate CVAs due to the impact of chronic cerebral hypoperfusion and redistribution of cerebral blood supply, and structural alterations in patients with MMD may be underestimated.

Alterations in cortical gray matter in patients with MMD, which mainly involves the precentral, postcentral, cingulate, and insula areas, have been documented in previous morphological studies [9–11]. However, to the best of our knowledge, few reports [8, 9] have evaluated structural changes in subcortical gray matter in patients with MMD. The cerebral areas (including part of the basal ganglia region) supplied by the middle cerebral arteries (MCA) are the typical ischemic sites involved in MMD [12]. Meanwhile, the basal ganglia and thalamus volumes were found to be decreased in patients with cortical stroke in the MCA territory at their chronic phase [13]. Therefore, acute CVAs, long-term hypoperfusion, and/or remote neuronal degeneration may result in structural alterations in subcortical gray matter in patients with MMD, which have not yet been sufficiently investigated.

Pure cognitive or psychiatric impairments in adult patients with MMD are rare in the literature [5, 12]. A systematic review [14] showed that the incidence rate of cognitive impairment in MMD was approximately 30%, with moderate to severe disturbance across varied cognitive domains. Although psychiatric changes are usually not onset symptoms, recent cross-sectional studies [15, 16] found that depression, anxiety, and posttraumatic stress syndrome were common complications in MMD survivors and were correlated with neurological impairment [15]. Hippocampus atrophy is regarded as an imaging hallmark of dementia and is seen in a variety of neurodegenerative diseases and poststroke dementia patients [17]. The amygdala is a critical structure in threat processing, emotional conditioning, and physiological response orchestration [18]. Thus, it would be important to study whether the two areas are abnormal in patients with MMD.

In light of these previous studies, we attempted to achieve a systematic assessment of the subcortical gray matter morphological changes in adult patients with MMD using an automated volumetric segmentation method and to investigate the correlation between subcortical volume and angiographic changes of intracranial arteries in patients with MMD. In addition, a novel parcellation tool based on a probabilistic atlas built with ultrahigh resolution ex vivo MRI data [19, 20] was used to further segment the hippocampus and the amygdala to observe the role of structural variations in the hippocampal subfields and the amygdala subnuclei in patients with MMD.

## Materials and methods

### Subjects

In our study, 161 patients from the Department of Neurosurgery who had been diagnosed with MMD via DSA were enrolled as potential participants. The inclusion criteria for patients included meeting the diagnostic criteria of the Research Committee on Moyamoya Disease of the Ministry of Health and Welfare (Japan, 1997), aged 18 ~ 60 years, not having a brain hemorrhage, not currently or previously receiving any antipsychotic drug treatments, and not having a history of revascularization operation. The patients with MMD recruited in our study were preoperatively recruited, which excluded the neuroprotective effects of revascularization and enabled a direct evaluation of underlying state-related abnormalities. The exclusion criteria included no history of psychiatric disorders, alcohol abuse, substance dependence, or any contraindications of MRI. A total of 142 patients (64 females) were finally included.

We also recruited 142 age- and sex-matched normal controls (NCs) (70 females) who had no history of drug dependence, psychiatric disorder, neurological disorder, or chronic medical disorder (such as heart failure). The average age of NCs was 37.01 years (SD = 10.23).

All participants were right-handed and were 18 to 60 years old. Informed consent was obtained from each individual participant included in the study. All procedures performed in this study were in accordance with the ethical standards of the Medical Ethics Committee of our hospital and with the 1964 Helsinki declaration and its later amendments or comparable ethical standards. The protocol was approved by the Medical Ethics Committee of our hospital.

### MRI acquisition

A 3.0-Tesla MAGNETOM Skyra MRI scanner (Siemens AG, Erlangen, Germany) equipped with a 20-channel phase-array head coil was used to acquire 3-dimensional high-resolution structural images. Every subject was placed in a supine position and was asked to keep his or her head as still as possible during MRI acquisition. The following magnetization-prepared 2 rapid gradient echoes (MP2RAGE) acquisition parameters were used: repetition time (TR) = 4000 ms; echo time (TE) = 2.98 ms; inversion time (TI) = 0 ms; flip angle = 0°; field of view (FOV) = 256 × 256 mm; slice thickness = 1 mm; number of slices = 176; and voxel size = 1 mm × 1 mm × 1 mm.

An additional 3-dimensional time-of-flight MRA was carried out after the structural scan using the following parameters: TR = 20 ms; TE = 3.43 ms; FA = 18°; FOV = 640 × 580 mm; and slice thickness = 0.5 mm.

### MRI analysis

We first investigated whether the structural images were impacted by head movement. The qualified images were automatically processed by FreeSurfer software (version 7.2.0, Massachusetts General Hospital, Boston, MA, U.S., http://surfer.nmr.mgh.harvard.edu) following the postprocessing stream comprehensively described by previous studies [19–22]. The reconstructed subcortical parcellation was visually checked to identify whether they followed gray/white matter boundaries and intensity borders, and if they were not aligned, each deviation was manually corrected for proper segmentation.

The volumes of subcortical gray matter, including the thalamus, caudate, putamen, pallidum, hippocampus, amygdala, and nucleus accumbens, were extracted. Furthermore, the hippocampus and the amygdala are respectively composed of interconnected heterogeneous subregions that relay different inputs from and outputs to multiple brain areas and contribute to distinct roles in cerebral function and disease processes. The segmentation of the hippocampus includes the parasubiculum, presubiculum, subiculum, cornu ammonis (CA)1, CA3, CA4, granule cell and molecular layer of the dentate gyrus (GC-ML-DG), molecular layer of the hippocampus (ML), hippocampus amygdala transition area (HATA), fimbria, hippocampal tail, and hippocampal fissure, and the CA1, CA3, CA4, GC-ML-DG, ML, presubiculum, and subiculum are further subdivided into head and body [20]. The amygdala is segmented into the anterior amygdaloid area (AAA), cortico-amygdaloid transition area (CAT), lateral nucleus, basal nucleus, paralaminar nucleus (PL), accessory basal nucleus (AB), medial nucleus, central nucleus, and cortical nucleus [19].

The degree of stenosis/occlusion of intracranial arteries including bilateral internal carotid arteries, anterior cerebral arteries, middle cerebral arteries, and posterior cerebral arteries in patients with MMD was evaluated on MRA images. The criteria we used were introduced by Houkin et al. [23], and the maximum possible MRA score for the unilateral side was 10. The MRA scores were allocated by two experienced neuroradiologists who were blinded to the clinical information.

### Statistical analysis

All statistical analyses were performed using SPSS 22.0 software (IBM Inc., Armonk, New York, U.S.). To assess the differences in volumes of subcortical gray matter between the patients with MMD and NCs, the Mann‒Whitney *U* test was performed. Correlations between the clinical/radiological features and gray matter volumes of the patients with MMD were analyzed using Spearman rank order correlation analysis.

The false discovery rate (FDR) correction was applied to between-group analyses and correlation analyses that involved multiple comparisons, and FDR-corrected *p* values < 0.05 were considered significant**.**

## Results

### Participant characteristics

The demographic information and clinical and radiological features of the patients with MMD are presented in Table 1. No significant differences in age or sex were detected between NCs and patients with MMD (*p* > 0.05). Some patients with MMD showed only mild symptoms (i.e., headache) (7/142, 4.93%), while many patients with MMD showed 2 or more kinds of signs. The most common symptom was motor deficits (109/142, 76.76%), followed by speech abnormality (aphasia or dysarthria) (42/142, 29.58%), cognitive impairment (22/142, 15.49%), and visual dysfunction (15/142, 10.56%). Notably almost half of the patients with motor deficits had concomitant sensory disturbance (52/109, 47.71%). No significant difference between MRA scores in the left and right hemispheres was found in patients with MMD (*p* > 0.05).

**Table 1:** Clinical and radiological features of patients with moyamoya disease

Characteristic	MMD (*n* = *142*)
Clinical features	
Age, years	37.2 ± 9.5
Sex, female:male	64:78
Duration of MMD, years	3.5 ± 5.7
Motor deficits	109 (76.8%)
Motor deficits without hemisensory disturbance	57 (40.1%)
Motor deficits with hemisensory disturbance	52 (36.6%)
Speech abnormity	42 (29.6%)
Cognitive impairment	22 (15.5%)
Visual dysfunction	15 (10.6%)
Headache	7 (4.9%)
Intracranial arteries features	
MRA scores of LH	5.1 ± 2.3
MRA scores of RH	5.2 ± 2.3

Abbreviations: MMD, moyamoya disease; LH, left hemisphere; RH, right hemisphere

Continuous variables are expressed as the means ± SD

### Subcortical gray matter volume

**Fig. 1:**
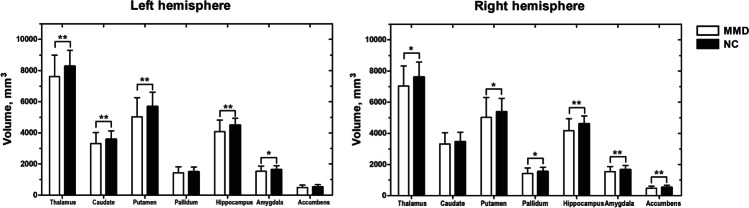
Comparison results of subcortical volume between the patients with moyamoya disease (MMD) and normal controls (NCs) (FDR-corrected *p* value < 0.05. The error bars indicate the standard deviation)

The comparisons of subcortical volume between groups are shown in Fig. 1. The Mann‒Whitney *U* test showed that compared with NCs, patients with MMD had significantly lower subcortical volumes in the bilateral thalamus, left caudate, bilateral putamen, bilateral hippocampus, bilateral amygdala, right pallidum, and right nucleus accumbens (FDR-corrected *p* value < 0.05).

### Volume of hippocampal subfields

Table 2 shows volumes of hippocampal subfields. Patients with MMD showed significantly lower volumes than the NCs in the bilateral CA1 body, bilateral CA1 head, bilateral CA3 head, bilateral CA4 body, bilateral CA4 head, bilateral fimbria, bilateral GC-ML-DG body, bilateral GC-ML-DG head, bilateral HATA, bilateral hippocampal fissure, bilateral ML body, bilateral ML head, left presubiculum head, left subiculum body, and left subiculum head (FDR-corrected *p* value < 0.05).

**Table 2:** Comparison results of the volume of hippocampal subfields between normal controls and patients with moyamoya disease

Hippocampal subfields	MMD	NC	*Z*	*p*
Left hemisphere				
CA1 body	122.2 ± 25.0	130.8 ± 20.9	− 3.36	< 0.001*
CA1 head	519.8 ± 94.6	562.3 ± 72.6	− 3.97	< 0.001**
CA3 body	90.7 ± 21.0	93.3 ± 14.8	− 1.18	0.240
CA3 head	123.3 ± 27.1	133.9 ± 20.1	− 3.83	< 0.001**
CA4 body	122.5 ± 23.1	128.6 ± 15.1	− 2.25	0.024*
CA4 head	129.0 ± 25.8	140.2 ± 18.6	− 4.06	< 0.001**
Fimbria	82.3 ± 22.7	97.7 ± 21.4	− 5.34	< 0.001**
GC-ML-DG body	137.6 ± 25.9	146.6 ± 16.7	− 3.14	0.0027*
GC-ML-DG head	155.1 ± 31.6	169.9 ± 22.7	− 4.39	< 0.001**
HATA	56.9 ± 13.5	63.3 ± 10.0	− 4.40	< 0.001**
Hippocampal fissure	158.2 ± 34.4	147.8 ± 26.6	− 2.96	0.003*
Hippocampal tail	579.2 ± 104.4	586.4 ± 76.8	− 0.26	0.796
ML body	229.9 ± 42.5	244.0 ± 27.1	− 2.94	0.003*
ML head	333.9 ± 60.7	362.8 ± 42.9	− 4.27	< 0.001**
Parasubiculum	67.2 ± 17.1	67.0 ± 11.5	− 0.24	0.808
Presubiculum body	178.3 ± 42.1	178.2 ± 30.4	− 0.89	0.373
Presubiculum head	144.3 ± 27.6	154.2 ± 19.4	− 2.81	0.005*
Subiculum body	255.2 ± 49.1	271 ± 36.6	− 2.44	0.015*
Subiculum head	194.8 ± 38.8	209.8 ± 30.9	− 3.15	0.002*
Right hemisphere				
CA1 body	134.7 ± 29.0	142.9 ± 22.8	− 2.93	0.003*
CA1 head	540.2 ± 93.3	577.8 ± 75.5	− 3.12	0.002*
CA3 body	99.9 ± 21.9	104.6 ± 18.1	− 1.54	0.125
CA3 head	127.9 ± 24.5	137.3 ± 20.7	− 3.31	< 0.001*
CA4 body	125.0 ± 23.6	134.2 ± 17.1	− 2.89	0.004*
CA4 head	133.1 ± 24.0	142.5 ± 18.2	− 3.23	0.001*
Fimbria	72.3 ± 22.6	91.8 ± 19.3	− 7.10	< 0.001**
GC-ML-DG body	139.4 ± 25.5	150.5 ± 18.1	− 3.28	0.001*
GC-ML-DG head	159.6 ± 29.3	172.8 ± 22.2	− 3.87	< 0.001**
HATA	53.9 ± 11.3	61.7 ± 9.8	− 5.76	< 0.001**
Hippocampal fissure	164.6 ± 39.3	150.0 ± 27.1	− 3.58	< 0.001*
Hippocampal tail	606.3 ± 109.2	605.8 ± 87.3	− 1.04	0.297
ML body	237.8 ± 43.6	253.1 ± 29.8	− 3.14	0.002*
ML head	345.2 ± 61.3	368.2 ± 44.1	− 2.79	0.005*
Parasubiculum	65.4 ± 16.5	63.0 ± 11.7	− 1.53	0.127
Presubiculum body	165.3 ± 41.8	163.4 ± 27.6	− 0.39	0.696
Presubiculum head	144.5 ± 29.3	149.4 ± 19.0	− 1.00	0.319
Subiculum body	254.0 ± 47.2	265.8 ± 36.7	− 1.67	0.095
Subiculum head	201.8 ± 42.2	209.5 ± 31.1	− 0.89	0.374

Abbreviations: CA, cornu ammonis; GC-ML-DG, granule cell and molecular layer of the dentate gyrus; ML, molecular layer of the hippocampus; HATA, hippocampus-amygdala-transition-area; MMD, moyamoya disease; NC, normal control

Continuous variables are expressed as the means ± SD

* FDR-corrected *p* value < 0.05

** FDR-corrected *p *value < 0.001

### Volume of the amygdala subnuclei

The MMD group showed significantly lower volumes than the NC group in the left AB, bilateral AAA, left lateral nucleus, and bilateral medial nucleus (FDR-corrected *p* value < 0.05) (Table 3).

**Table 3:** Comparison results of the volume of the amygdala subnuclei between normal controls and patients with moyamoya disease

The amygdala subnuclei	MMD	NC	*Z*	*p*
Left hemisphere				
AB	275.8 ± 53.7	290.3 ± 35.8	− 2.32	0.020*
AAA	57.9 ± 10.7	62.4 ± 8.2	− 3.64	< 0.001*
Basal nucleus	462.9 ± 82.5	481.9 ± 55.2	− 1.73	0.084
Central nucleus	45.6 ± 12.5	45.2 ± 8.8	− 0.39	0.696
Cortical nucleus	29.0 ± 7.1	29.5 ± 5.0	− 0.01	0.992
CAT	191.7 ± 35.4	198.2 ± 25.1	− 1.34	0.181
Lateral nucleus	656.1 ± 107.7	693.3 ± 82.0	− 2.57	0.010*
Medial nucleus	25.7 ± 7.8	23.8 ± 6.3	− 2.53	0.011*
PL	53.8 ± 10.1	54.5 ± 6.5	− 0.30	0.767
Right hemisphere				
AB	277.5 ± 54.4	292.0 ± 38.5	− 1.75	0.081
AAA	60.7 ± 11.3	65.6 ± 8.5	− 3.72	< 0.001*
Basal nucleus	467.3 ± 83.8	486.7 ± 59.0	− 1.48	0.14
Central nucleus	50.1 ± 12.7	48.6 ± 9.4	− 2.12	0.034
Cortical nucleus	28.9 ± 7.2	29.2 ± 4.9	− 0.20	0.845
CAT	182.2 ± 33.2	191.2 ± 25.0	− 1.91	0.056
Lateral nucleus	681.8 ± 112.6	712.1 ± 81.7	− 1.83	0.068
Medial nucleus	26.5 ± 8.2	24.5 ± 6.3	− 2.76	0.006*
PL	52.3 ± 9.6	53.1 ± 6.5	− 0.04	0.967

Abbreviations: AAA, anterior amygdaloid area; AB, accessory basal nucleus; CAT, cortico-amygdaloid transition area; MMD, moyamoya disease; NC, normal control; PL, paralaminar nucleus

Continuous variables are expressed as the means ± SD

* FDR-corrected *p* value < 0.05

### Correlation analysis

Correlations among the subcortical volumes and clinical/radiological measurements of patients with MMD are displayed in Supplementary Tables 1–3 (FDR-corrected *p* value < 0.05).

The duration of MMD showed significant negative correlations with volumes in the bilateral thalamus, putamen, hippocampus, amygdala, and right caudate (see Table S1). For hippocampal subfields, durations of MMD showed significant negative correlations with volumes in the bilateral CA1 head, right CA1 body, right CA4 body, right fimbria, left GC-ML-DG head right GC-ML-DG body, bilateral ML head, right ML body, bilateral presubiculum head, left presubiculum body, bilateral subiculum head, and right subiculum body (see Table S2). The duration of MMD displayed significantly negative correlations with volumes in all amygdala subnuclei except the left AAA (see Table S3).

Significantly negative correlations were found between the MRA scores and volumes in the left caudate, bilateral putamen, bilateral pallidum, left amygdala, bilateral nucleus accumbens (see Table S1); left CA1 body, left CA1 head, left CA3 head, left CA4 head, left fimbria, left GC-ML-DG body, left GC-ML-DG head, bilateral HATA, left hippocampal tail, left ML body, left ML head, left parasubiculum, left presubiculum body, left presubiculum head, left subiculum body, left subiculum head (see Table S2); left AB, left basal nucleus, left central nucleus, left cortical nucleus, left CAT, left lateral nucleus, left medial nucleus, and left PL (see Table S3).

In addition, the duration of MMD was positively related to the MRA score of the left hemisphere (*r* = 0.169, *p* = 0.045), and tended to be positively related to the MRA score of the right hemisphere (*r* = 0.150, *p* = 0.076).

## Discussion

The present study sought to investigate the structural patterns of subcortical gray matter volume in adult patients with MMD, and several novel findings were revealed. The bilateral thalamus, left caudate, bilateral putamen, bilateral hippocampus, bilateral amygdala, right pallidum, and right nucleus accumbens showed significant volumetric decreases in patients with MMD. Widespread hippocampal subregions and several amygdala subnuclei in patients with MMD were found to be shrunken, and morphological alterations in specific subregions were more obvious than in the whole hippocampus/amygdala. These structural alterations in patients with MMD were correlated with disease duration and intracranial arterial changes, suggesting a probable linkage with chronic hemodynamic pathogenesis.

Based on the frontal-subcortical framework, the subcortical gray matter plays crucial roles in sensorimotor control as well as in a wide array of nonmotor functions ranging from cognitive to affective-motivational behavioral processing [24]. More than three-fourths of patients with MMD enrolled in this study experienced motor deficits, which have been primarily implicated in structural and functional abnormalities in the primary motor cortex [2, 10]. The basal ganglia are core components of the extrapyramidal motor system and have a fundamental and distinctive role in the control of normal motor outputs. The striatum is the central input nucleus of the basal ganglia. The dorsal striatum (caudate-putamen), which receives projections from the motor cortex, participates in body movement execution, while the ventral striatum (the nucleus accumbens) can modulate locomotion activity through limbic information [25]. Structural impairments of the basal ganglia have been demonstrated to be related to extrapyramidal diseases, including Parkinson’s disease (PD) [26] and Huntington’s disease (HD) [27], as well as cerebrovascular diseases, such as stroke [13]. Descriptions of the clinical course of movement disorders in MMD have usually been ignored in the published literature; however, according to a systematic questionnaire study in European MMD [28], up to 55% of the participants had a history of movement disorders. The underlying pathophysiologic mechanism and clinical implications of secondary movement disorders in MMD warrant further investigation.

The thalamus is considered as a relay simply transferring information from the periphery to the neocortex or one cortical/subcortical region to another; however, accumulating evidence indicates that the thalamus also plays a key role in higher-order cognition, such as learning and memory, shaping mental representations, and flexible adaptation [29]. Recent neuroimaging studies have provided further support for the involvement of the basal ganglia in aspects of working memory, executive function, cognitive flexibility, and response interference control [30–33]. In the present study, volumetric decreases in subcortical areas were observed across several regions, but only 15.49% of patients with MMD manifested significant cognitive impairments. Considering the high proportion of motor deficits in our study, this paradoxical result suggests that subcortical atrophy in MMD may be mainly correlated with motor deficits, or the clinical assessment of cognitive decline in patients with MMD may be far from adequate. The significant hippocampal atrophy in MMD makes the latter explanation more reasonable since direct infarction in the hippocampus is rare [34], and primary infarcted regions can lead to remote hippocampal neurodegeneration due to decreases in functional and structural connections between infarcted regions and the hippocampus, resulting in cognitive impairment [35].

As a hallmark related to neurodegenerative diseases, the hippocampus is critical for memory acquisition, consolidation, and retrieval, and is involved in episodic and semantic long-term memory [36]. The importance of hippocampal subfields as a sensitive biomarker for the early detection of dementia has been gradually realized. Different subtypes of dementia have been found to exhibit specific atrophy of hippocampal subfields [17, 34, 37]. In our study, different hippocampal subregions and even different parts of one hippocampal subregion (head and body) showed distinct volumetric vulnerability. Each hippocampal subregion has unique projections to specific cerebral regions that form different hippocampal loops and has unique specialization for hippocampal processing and memory [35]; therefore, it appears likely that the overall volume of the hippocampus is unreliable or unstable, and the subregion volumes contain specific information in addition to the total hippocampal volume. For instance, the CA1 hippocampal neurons are most vulnerable to neurodegeneration from acute pathological conditions such as ischemia, hypoglycemia, and epileptic attack, while the DG or the CA3 tends to show evident damage after chronic insults such as chronic epilepsy, chronic ischemia, and neuropsychiatric disorders [36]. Atrophy of the CA1, CA3, and DG was found in patients with MMD in our study, indicating that both acute CVAs and chronic cerebral hemodynamic changes may be attributed to hippocampal anatomical deficits.

Similar to the hippocampus, different subregions of the amygdala have distinct levels of vulnerability to neuronal impairments correlated with MMD. Unlike the hippocampus, the range and degree of morphological changes in the amygdala subnuclei were less serious. This may reflect that pathological mechanisms underlying the morphological impairments were different between the amygdala and the hippocampus in patients with MMD.,Psychological presentations of MMD generally receive little attention from neurosurgeons/neurologists and even the patients themselves. With the lack of an effective remedy, while suffering a long-term course of the disease, and worrying about the uncertain risk of CVAs, more than a quarter of adult patients with MMD may experience psychological comorbidities such as depression and anxiety [1]. Recent depression studies investigating volumes of the amygdala found that amygdala volume increases in the early stage of depression [38], and as the duration developes over time, the volume of the amygdala declines [39], suggesting that the dynamic characteristics of amygdala volume may differ depending on the developmental stages of depression. Additionally, hippocampal atrophy may lead to abnormal morphological changes in the amygdala due to reciprocal projections. Although patients with MMD recruited in our study did not receive any objective assessment of depression or anxiety, we speculate that the observed volume decrease in the amygdala, which was related to MRA scores, seems to be a multifactorial result of chronic cerebral hemodynamic insufficiency, related secondary neurodegeneration, and mental stress.

With greater disease duration or angiographic changes in the intracranial artery, subcortical areas in MMD showed smaller volumes, indicating chronic cerebral hemodynamic progression underlying the morphological impairments. However, the duration could not precisely reflect the accurate time of MMD involvement because a considerable proportion of the patients in our study were diagnosed with MMD for only several months at the time of MRI scanning. Taking a rare case as an example, a 41-year-old man with sudden hypoesthesia and headache was diagnosed with postinfectious Moyamoya syndrome, which was thought to be related to a history of meningitis 2 years ago [40]. However, most patients with MMD do not have the exact etiologies and time of onset. The CVAs were induced by hemodynamics from the MMD itself, and subcortical morphology might have already been compromised at the time of the CVAs occurring. Therefore, the observed correlations between the duration of MMD and structural changes in subcortical areas could not truly reflect the whole situation. An interesting finding is that altered subcortical volumes showed more correlations with the MRA scores of the left hemisphere, though the MRA scores in MMD did not display a significant difference between the bilateral hemispheres. Meanwhile, only the MRA score of the left hemisphere showed a positive correlation with the duration of MMD. One possible explanation for these phenomena is that neurological traits such as functional asymmetry and unbalanced hemispheric interaction may be implicated in pathological processing.

The primary strength of this study is the large sample size with a relatively balanced sex distribution. To the best of our knowledge, this is the first study to systematically examine morphological changes in subcortical areas, hippocampal subfields, and amygdala subnuclei in adult patients with MMD. Several limitations and possible improvement suggestions should be acknowledged. First, this was a cross-sectional design study. Longitudinal and follow-up patients are needed in further studies to provide an integrated view of the nature, rate, and possible causative mechanisms of subcortical volume loss in MMD. Second, the heterogeneous symptoms in patients with MMD may have led to potential symptom-specific morphological changes unrevealed. Third, the duration of illness within our sample varied, ranging from months to years, and some patients even suffered from MMD for more than a decade. Due to the limited ability of MRA in arterial visualization, the intracranial macrovascular characteristics of those chronic patients might tend toward stability, resulting in diminished correlations between MRA scores and altered regions. Fourth, several hippocampal subregions and amygdala subnuclei are too small, and the sensitivity to reliably delineate these areas may not be sufficient using 3 T magnetic field T1-weighted images with a relatively low signal-to-noise ratio. The volume of hippocampal subregions and the amygdala subnuclei must thus be interpreted with caution. Fifth, although MRA is a noninvasive, reliable diagnostic tool for MMD, the gold standard for vessel assessment is DSA, and signal intensity close to stenosis could be lost in TOF images due to slowing/turbulence of the blood flow, which could be a bias. Finally, this study neglected the evaluation of neuropsychological states due to insufficiently detailed interrogation and inadequate physical examination, resulting in an underestimation of cognitive and emotional changes in MMD. Detailed and specialized cognitive and psychiatric assessments in further studies might be useful for obtaining more robust results, and comprehensive screening for neuropsychological changes in MMD might be beneficial to determine vulnerable individuals who might need timely and effective interventions with reperfusion protocols, which may repair cerebral function, and probably prevent new CVA onset.

## Conclusion

Our study observed volumetric atrophy of the thalamus, caudate, putamen, hippocampus, amygdala, pallidum, and nucleus accumbens in patients with MMD. The subregion volume analysis demonstrated a more detailed understanding of the pathophysiology in MMD by revealing distinct patterns of volumetric vulnerability in hippocampal subfields and amygdala subnuclei, implying potential cognitive and affective impairments in MMD, which should be carefully recognized by clinicians and discreetly evaluated in the process of disease management. In addition, disease durations and intracranial arterial changes were correlated with several subcortical volume reductions in patients with MMD. These findings provide new evidence of subcortical volume alterations as a potential biomarker that provides supplementary information for screening practices, intervention planning, and prognostic assessment of MMD. Future studies are suggested to identify whether subcortical atrophy is related to functional changes and neuropsychological impairments in patients with MMD.

## Supplementary Information

Below is the link to the electronic supplementary material.

Supplementary file1 (DOCX 33 KB)
