# Supplementary material for: Atrophy in subcortical gray matter in adult patients with moyamoya disease
Source: Neurol Sci. 2023 Jan 9;44(5):1709–17. doi: 10.1007/s10072-022-06583-x (PMC10102099; doi:10.1007/s10072-022-06583-x)
Supplement: Supplementary file 1 — Supplementary file1 (DOCX 33 KB) [file 10072_2022_6583_MOESM1_ESM.docx]

**Table S1. Correlations among the subcortical gray matter volumes, disease duration, and MRA scores in patients with moyamoya disease.**

| Subcortical area | Disease duration | |  | MRA score | |
| --- | --- | --- | --- | --- | --- |
|  | *r* | *p* |  | *r* | *p* |
| Left thalamus | -0.18 | 0.032* |  | -0.15 | 0.081 |
| Left caudate | -0.13 | 0.115 |  | -0.24 | 0.003* |
| Left putamen | -0.19 | 0.028* |  | -0.32 | < 0.001** |
| Left pallidum | -0.19 | 0.021 |  | -0.32 | < 0.001** |
| Left hippocampus | -0.22 | 0.009* |  | -0.15 | 0.071 |
| Left amygdala | -0.18 | 0.034* |  | -0.36 | < 0.001** |
| Left nucleus accumbens | -0.12 | 0.144 |  | -0.31 | < 0.001** |
| Right thalamus | -0.19 | 0.022* |  | 0.02 | 0.818 |
| Right caudate | -0.23 | 0.006* |  | -0.18 | 0.035 |
| Right putamen | -0.22 | 0.008* |  | -0.25 | 0.003* |
| Right pallidum | -0.11 | 0.206 |  | -0.21 | 0.011* |
| Right hippocampus | -0.26 | 0.002* |  | -0.06 | 0.461 |
| Right amygdala | -0.22 | 0.010* |  | -0.15 | 0.076 |
| Right nucleus accumbens | -0.11 | 0.213 |  | -0.30 | < 0.001* |

Note: * FDR-corrected *p* value < 0.05.

** FDR-corrected *p* value < 0.001.

**Table S2. Correlations among the volumes of hippocampal subfields, disease duration, and MRA scores in patients with moyamoya disease.**

| Hippocampal subfields | Disease duration | |  | MRA score | |
| --- | --- | --- | --- | --- | --- |
|  | *r* | *p* |  | *r* | *p* |

| Left CA1 body | -0.10 | 0.247 |  | -0.18 | 0.028* |
| --- | --- | --- | --- | --- | --- |
| Left CA1 head | -0.20 | 0.016* |  | -0.25 | 0.002* |
| Left CA3 body | 0.01 | 0.905 |  | 0.07 | 0.383 |
| Left CA3 head | -0.14 | 0.091 |  | -0.25 | 0.002* |
| Left CA4 body | -0.14 | 0.093 |  | -0.17 | 0.049 |
| Left CA4 head | -0.19 | 0.024 |  | -0.29 | 0.001* |
| Left fimbria | -0.15 | 0.073 |  | -0.23 | 0.005* |
| Left GC-ML-DG body | -0.14 | 0.110 |  | -0.21 | 0.011* |
| Left GC-ML-DG head | -0.20 | 0.018* |  | -0.31 | < 0.001* |
| Left HATA | -0.11 | 0.187 |  | -0.26 | 0.002* |
| Left hippocampal tail | -0.28 | 0.001 |  | -0.27 | 0.001* |
| Left hippocampal fissure | 0.04 | 0.607 |  | 0.05 | 0.588 |
| Left ML body | -0.18 | 0.035 |  | -0.25 | 0.003* |
| Left ML head | -0.24 | 0.003* |  | -0.30 | < 0.001* |
| Left parasubiculum | -0.15 | 0.083 |  | -0.25 | 0.002* |
| Left presubiculum body | -0.22 | 0.007* |  | -0.38 | < 0.001** |
| Left presubiculum head | -0.30 | < 0.001* |  | -0.31 | < 0.001* |
| Left subiculum body | -0.16 | 0.066 |  | -0.23 | 0.006* |
| Left subiculum head | -0.23 | 0.007* |  | -0.18 | 0.031* |
| Right CA1 body | -0.19 | 0.024* |  | -0.13 | 0.112 |
| Right CA1 head | -0.22 | 0.009* |  | -0.12 | 0.155 |
| Right CA3 body | -0.14 | 0.088 |  | 0.03 | 0.738 |
| Right CA3 head | -0.11 | 0.213 |  | -0.17 | 0.047 |
| Right CA4 body | -0.24 | 0.004* |  | -0.04 | 0.606 |
| Right CA4 head | -0.15 | 0.079 |  | -0.15 | 0.068 |
| Right fimbria | -0.20 | 0.019* |  | -0.15 | 0.081 |
| Right GC-ML-DG body | -0.24 | 0.005* |  | -0.07 | 0.437 |
| Right GC-ML-DG head | -0.18 | 0.033 |  | -0.18 | 0.031 |
| Right HATA | -0.14 | 0.092 |  | -0.29 | < 0.001* |
| Right hippocampal tail | -0.18 | 0.037 |  | -0.08 | 0.345 |
| Right hippocampal fissure | 0.09 | 0.272 |  | 0.13 | 0.112 |
| Right ML body | -0.28 | 0.001* |  | -0.15 | 0.079 |
| Right ML head | -0.25 | 0.003* |  | -0.12 | 0.145 |
| Right parasubiculum | -0.22 | 0.010* |  | -0.12 | 0.146 |
| Right presubiculum body | -0.18 | 0.037 |  | -0.15 | 0.072 |
| Right presubiculum head | -0.31 | < 0.001* |  | -0.13 | 0.133 |
| Right subiculum body | -0.19 | 0.023* |  | -0.05 | 0.539 |
| Right subiculum head | -0.24 | 0.004* |  | -0.04 | 0.635 |

Abbreviations: CA, cornu ammonis; GC-ML-DG, granule cell and molecular layer of the dentate gyrus; ML, molecular layer of the hippocampus; HATA, hippocampus-amygdala-transition-area.

* FDR-corrected *p* value < 0.05.

** FDR-corrected *p* value < 0.001.

**Table S3. Correlations among the volumes of the amygdala subnuclei, disease duration, and MRA scores in patients with moyamoya disease.**

| The amygdala subnuclei | Disease duration | |  | MRA score | |
| --- | --- | --- | --- | --- | --- |
|  | *r* | *p* |  | *r* | *p* |
| Left AB | -0.28 | 0.001* |  | -0.29 | < 0.001* |
| Left AAA | -0.11 | 0.204 |  | -0.17 | 0.050 |
| Left basal nucleus | -0.23 | 0.007* |  | -0.22 | 0.008* |
| Left central nucleus | -0.24 | 0.005* |  | -0.24 | 0.005* |
| Left cortical nucleus | -0.32 | < 0.001* |  | -0.33 | < 0.001** |
| Left CAT | -0.23 | 0.007* |  | -0.27 | 0.001* |
| Left lateral nucleus | -0.18 | 0.029* |  | -0.25 | 0.003* |
| Left medial nucleus | -0.25 | 0.003* |  | -0.27 | 0.001* |
| Left PL | -0.17 | 0.038* |  | -0.21 | 0.014* |
| Right AB | -0.28 | 0.001* |  | -0.19 | 0.028 |
| Right AAA | -0.17 | 0.044* |  | -0.12 | 0.143 |
| Right basal nucleus | -0.21 | 0.012* |  | -0.12 | 0.164 |
| Right central nucleus | -0.29 | 0.001* |  | -0.19 | 0.020 |
| Right cortical nucleus | -0.33 | < 0.001** |  | -0.11 | 0.186 |
| Right CAT | -0.26 | 0.002* |  | -0.16 | 0.052 |
| Right lateral nucleus | -0.19 | 0.023* |  | -0.11 | 0.183 |
| Right medial nucleus | -0.26 | 0.002* |  | -0.14 | 0.107 |
| Right PL | -0.18 | 0.036* |  | -0.06 | 0.480 |

Abbreviations: AAA, anterior amygdaloid area; AB, accessory basal nucleus; CAT, cortico-amygdaloid transition; PL, paralaminar nucleus.

* FDR-corrected *p* value < 0.05.

** FDR-corrected *p* value < 0.001.
